# Effect of occipitocervical fusion with screw-rod system for upper cervical spine tumor

**DOI:** 10.1186/1471-2482-14-30

**Published:** 2014-05-18

**Authors:** Jun Zou, Chenxi Yuan, Ruofu Zhu, Zhiming Zhang, Weimin Jiang, Huilin Yang

**Affiliations:** 1 Department of Orthopaedic Surgery, The First Affiliated Hospital of Soochow University, 188 Shizi St, Suzhou, Jiangsu 215006, China

**Keywords:** Occipitocervical fusion, Upper cervical spine, Tumor, Reconstruction

## Abstract

**Background:**

Craniospinal junction tumors are rare but severe lesions. Surgical stabilization has been established to be an ideal treatment for upper cervical tumor pathology. The purpose of this study was to evaluate the effect of a screw-rod system for occipitocervical fusion.

**Methods:**

A total of 24 cases with C1 and C2 cervical tumor underwent occipitocervical fusion with Vertex screw-rod internal fixation from January 2005 to December 2012. Preoperative X-ray and MRI examinations were performed on all patients before the operation, after the operation, and during last follow-up. The JOA score was used to assess neurological function pre and postoperatively.

**Results:**

All the patients were followed up for 6 to 42 months with an average of 24 months. The result of X-ray showed that bony fusion was successful in 18 patients at 3 months and 6 patients at 6 months of follow-ups. There was no deterioration of spinal cord injury. The JOA Scores of neurological function increased significantly.

**Conclusion:**

The screw-rod system offers strong fixation and good fusion for occipitocervical fusion. It is an effective and reliable method for reconstruction of upper cervical spine tumor.

## Background

Upper cervical spine tumor is a main cause of upper cervical spinal cord compression. The key points of its treatment are timely decompression, restoration of normal cervical anatomy and reconstruction of stability [1]. The treatment of these upper cervical spine tumors involving C1 and C2 segments has improved considerably in the last decades. Conventional approaches to posterior occipitocervical fixation and fusion after tumor resection, including bone plate-and-wire, rod-and-wire and occipitocervical plate, all have different shortcomings [2]. Recently, with improvement in internal fixation techniques and advances in surgical skill, many new internal fixation instruments have been available for occipitocervical fusion [3]. We carried out a retrospective study of 24 patients who received occipitocervical fusion with a screw-rod internal fixation system. The purpose was to investigate the therapeutic effect and clinical value of this screw-rod internal fixation system in occipitocervical fusion for reconstruction of upper cervical spine tumor.

## Methods

The study was approved by the Institutional Ethics Committee of The First Affiliated Hospital of Soochow University, and all patients provided both written and verbal informed consent for participation. A total of 24 patients with C1 and C2 cervical tumor were enrolled in this study (11 males and 13 females; the mean age: 48.4 years ranged from 32 to 62 years) and treated by Vertex screw-rod internal fixation system (Medtronic Inc., Minneapolis, MN, USA) in our hospital between January 2005 and December 2012. All patients exhibited occipitocervical pain, cervical weakness, difficulty in cervical rotation and symptoms caused by upper cervical tumor. 13 patients experienced neurological deficits as a consequence of medullary compression, such as weakness in the upper extremities, unsteady gait, hypermyotonia, and tendon hyperreflexia.

The head of each patient was fixed with a Mayfield skull clamp. Head and neck were maintained in the neutral position or slightly flexed. A posterior midline incision was made from the occipital protuberance to the next spinal process of the most distal cervical vertebra to be fused. Subperiosteal dissection was carried out to expose the posterior structure of the occipitocervical area. The occipital area was exposed 2 cm laterally, and the posterior atlantal arch was exposed no wider than 1.5 cm laterally. It should be exposed to the lateral margin of the lateral mass under the C2 level. And then, necessary resection of the lamina, articular process and tumor is carried out. The screw insertion site was located at the dorsal surface of the articular process, and grinded out the cortical bone with drill. After identifying the direction of screw insertion, the screw was placed into the pedicle with a hand-drill under the guidance of preoperative imaging and intraoperative C-arm monitoring. The medial wall of the pedicle was identified with a neural probe and tapped under direct visualization. After confirming with a probe that the circumference of the channel was bony structure, the suitable diameter pedicle screws were placed into the prepared channels. Bended and inserted adaptable rods, then tighten the nuts of screws. If the lamina was preserved, bone grafted posterolaterally. If the lamina was removed, cartilage surface of the facet joint at the level of fusion was chipped off by the rongeur, and cancellous bone fragments were implanted. Skull traction was removed immediately after the operation and neck collar fixation was applied. Patients started to sit and walk from the next day and the neck collar was applied for 6-8 weeks.

Preoperative X-ray, CT and MRI examinations were performed on all patients. The Japanese Orthopaedic Association (JOA) score was used to assess neurological function pre and postoperatively. JOA score has been accepted universally as a tool to measure the outcomes of surgical and nonsurgical treatments for various cervical spinal disorders. It was determined by direct questioning to assess subjective symptoms, clinical signs, and restriction of activities of daily living. A decrease in the score represents deterioration of function, and an increase represents improvement. SPSS 14.0 (SPSS Inc., Chicago, IL, USA) was used to analyze the data. The paired sample *t*-test was used. Statistical significance was accepted for p values of p < 0.05.

## Results

We followed up all the patients: the mean duration of follow-up was 24 months, ranging from 6 to 42 months. In 18 patients, X-ray taken 3 months later showed successful occipitocervical fusions; in 6 patients, X-ray taken 6 months later showed successful occipitocervical fusions. There was no complication such as aggravation of the spinal cord injury. The mean preoperative JOA score was 12.2 points, ranging from 4 to 16 points, the mean postoperative JOA score was 16.8 points, ranging from 9 to 17 points, and the improvement was significant (p = 0.000). Partial loss of rotation function occurred in all patients. 100-degree rotation was achieved in 19 patients, 80-degree rotation was achieved in 4 patients, and 50-degree rotation was achieved in one patient.

**Figure 1:**
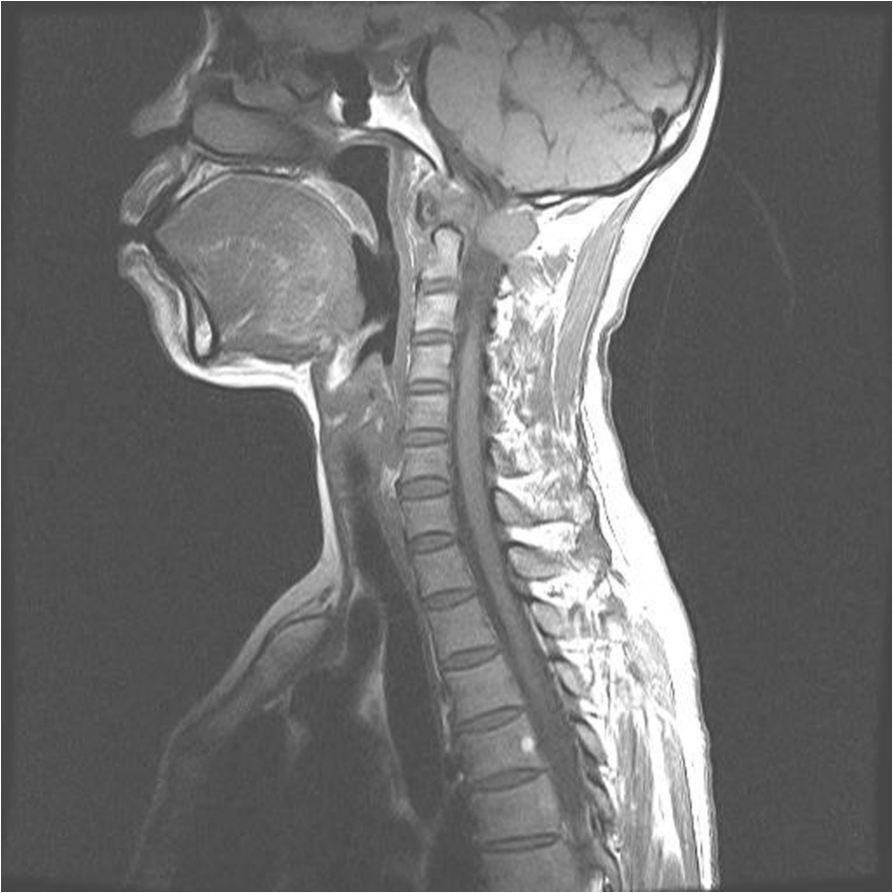
Sagital MRI preoperative image.

**Figure 2:**
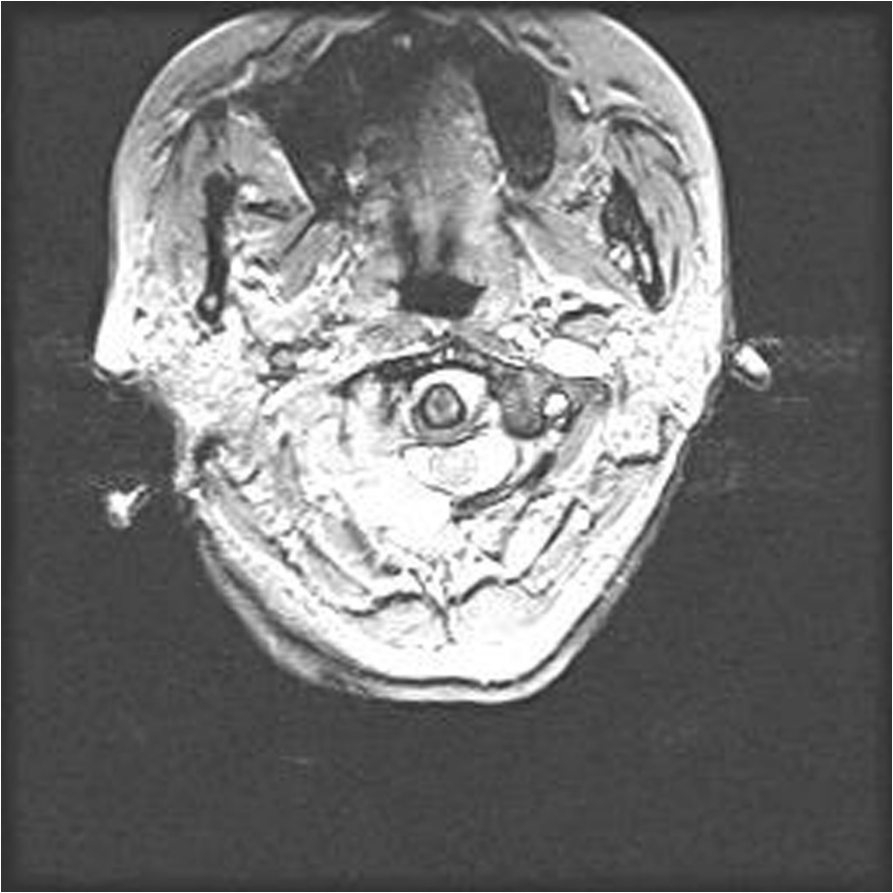
Axial MRI preoperative image.

**Figure 3:**
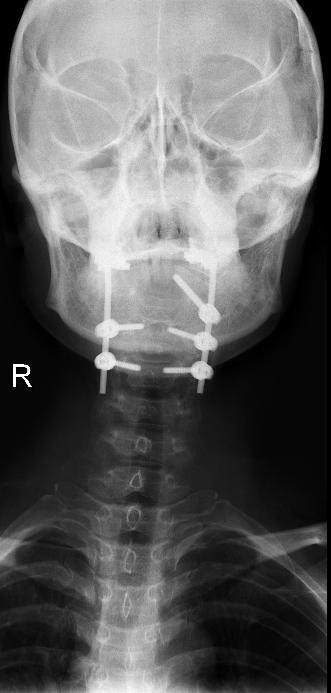
Anteroposterior X-ray postoperative image.

**Figure 4:**
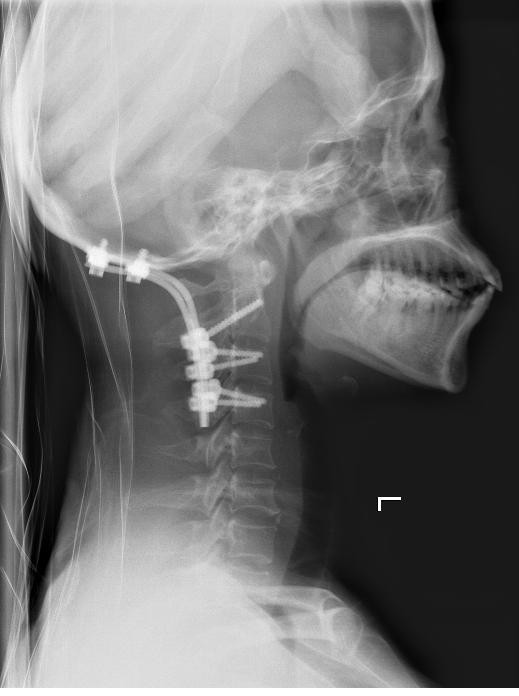
Lateral X-ray postoperative image.

Illustrative Case: A 54 year old female with upper cervical pain, and limitation of movement of the head (breast cancer metastasis). MRI scans showed a tumor that involved right C1 lateral mass. The tumor was completely resected through a posterior approach, and occipitocervical fixation from occiput to C4 was performed. The patient presented no postoperative pain and the spine was stable and aligned (Figures 1, 2, 3 and 4).

## Discussion

Occipitocervical fusion is a very effective method for reconstruction of upper cervical instability caused by tumor. Many fusion methods have been developed, such as conventional occipitocervical fusion, which adopts simple bone grafting for fusion. Foerster firstly proposed the occipitocervical fusion with free vascularized fibular grafting in 1927 [4]. After then, many different kinds of posterior occipitocervical fusion and fixation techniques have been reported. Occipitocervical bone grafting with additional sublaminar wiring may be more stable than single bone grafting. However, the patient still need bed rest for 12 weeks. Spinal cord injury and rotational instability often occurred in those patients [5]. Ransford described a novel technique in 1986. An anatomically contoured steel loop was secured to the occiput via small burr holes and to the vertebrae by sublaminar wiring. It afforded a rigid stabilization. The patient could mobilise with a cervical collar or cervicodorsal brace only [6]. In early 1990s, occipitocervical plate had been applied, and then screw-rod system. In 2000, universal vertebral pedicle screw has been widely used. The posterior occipitocervical fixation could provide more stable fixation, which is relatively easier to perform and less risky [7,8].

This screw-rod fixation system is comprised of cervical laminar hooks, thoracic multi-axial screws, and lateral offset connectors. The laminar hooks are designed to provide excellent fit and fixation to cervical spine. The multi-axial screw offers a degree of angulation and independent screw placement that reduces the need to contour the rod. The articulating saddle of the multi-axial screw allows for easy rod attachment. A lateral offset connector provides a way to connect non-linear multi-axial screws to the rod. These advantages are not only beneficial for intraoperative manipulation, providing strong internal fixation, but also favorable to the fusion achieved by bone graft and the maintenance of postoperative occipitocervical contour.

In our study, this system was carried out for all upper cervical spine tumor cases. However, screw-rod fixation at the upper cervical spine is relatively difficult during occipitocervical fusion. There are many papers describing the insertion point, direction and length of the cervical pedicle screw in detail. However, because of differences among individuals, sexes, and the nature of diseases, the anatomy of the pedicle and peripheral structures also differs. It is certain that there is no fixed standard for identifying the insertion site of the pedicle screw, and so we need to be flexible while selecting the site and direction of screw insertion and choosing a corresponding screw insertion plan for each individual. Our experience is that detailed imaging examinations should be performed before the operation including: anteroposterior, lateral, and both oblique views of X-ray and cervical CT. If the outer diameter of the axis is large, then the screw insertion can be performed after exploring the medial wall of pedicle during the operation. This method is quite accurate; however, because the outer diameters of lower cervical pedicles are small, screw insertion is difficult in these areas. After identifying the insertion site, our insertion method is similar to the method introduced by Abumi et al. [9].

According to anatomical observation of thickness distribution of the occipital bone, during the occipitocervical fusion, the optimal position of screw fixation in the occipital bone is the triangle area in which the thickness of the occipital bone is more than 8 mm. It is reported that the proper thickness of plate or bone graft during the occipitocervical fusion is 3 to 5 mm [10]. Therefore the length of screw in this area could be 8 to 12 mm; the ideal length of screw is 8 to 10 mm; and screws 10 to 14 mm in length should be applied in the external occipital protuberance. Heywood considered that the safest length of screw is 8 mm and the area with bone thickness more than 8 mm is the safest place for the screw insertion [11]. That is, screws 8 to 12 mm in length are usually applied in this area and screws less than 8 mm in length can be used in the adjacent area in which the bone thickness is 6 to 8 mm. This area is located about 1 cm inferior to the area where the bone thickness is more than 8 mm. However, the drilling depth should not exceed 6 mm in this area and for operation safety. The drilling point should be as close to the midline and nuchal line as possible to stay away from the fossa cerebellaris.

The main intraoperative complications of this technique are injuries of the spinal cord, nerve root and vertebral artery caused by pedicle perforation during drilling or screw insertion. Some papers report that cervical screw-rod fixation exhibits a relatively high perforation rate through the postoperative imaging examination and relatively low corresponding complications. Yoshimoto et al. reported that in 134 pedicle screws applied for fixation, postoperative imaging examination showed that 15 screws perforated the pedicle and the perforation rate was 11.1%, but there was no complication related to vascular and neural injuries [12]. Abumi et al. reported that in a total of 712 screws, 669 screws were evaluated with imaging examination and 45 screws perforated the pedicle. The perforation rate was 6.7%, but complications from vascular and neural injuries happened only in 3 cases [13]. And the long-term complication of occipitocervical fusion is fusion failure. In our study, screw insertion was successful during the operation and there was no injury of vertebral artery, spinal cord or nerve root. And there was no complication related to screw insertion after the operation and during the later follow-up period. Among all 24 patients in our study, 18 patients achieved successful occipitocervical fusions 3 months after the surgery, and 6 patients achieved successful fusions 6 months after the surgery.

## Conclusion

In conclusion, rigid occipitocervical screw-rod fixation system proved to be safe and beneficial to patients with upper cervical tumor. Long-term evaluation of the device in terms of the acquisition of bone fusion and spinal stabilization, as well as the recovery of neurologic deficit, is satisfactory. However, care in preoperative planning, placement of the screws, and contouring of the rod is still necessary.

## Competing interests

The authors declare that they have no competing interests.

## Authors’ contributions

All authors read and approved the final manuscript. JZ and HY concepted and designed the study; CY, RZ acquired the data; ZZ and WJ analyzed and interpreted the data; JZ and RZ drafted the manuscript; CY performed a statistical analysis; WJ and HY supervised the study.

## Pre-publication history

The pre-publication history for this paper can be accessed here:

http://www.biomedcentral.com/1471-2482/14/30/prepub
